# The Genetic Architectures of Functional and Structural Connectivity Properties within Cerebral Resting-State Networks

**DOI:** 10.1523/ENEURO.0242-22.2023

**Published:** 2023-04-07

**Authors:** Elleke Tissink, Josefin Werme, Siemon C. de Lange, Jeanne E. Savage, Yongbin Wei, Christiaan A. de Leeuw, Mats Nagel, Danielle Posthuma, Martijn P. van den Heuvel

**Affiliations:** 1 Department of Complex Trait Genetics, Center for Neurogenomics and Cognitive Research, Vrije Universiteit Amsterdam, Amsterdam Neuroscience, Amsterdam 1081 HV, The Netherlands; 2 Department of Sleep and Cognition, Netherlands Institute for Neuroscience, Amsterdam 1105 BA, The Netherlands; 3 School of Artificial Intelligence, Beijing University of Posts and Telecommunications, Beijing 100876, China; 4 Department of Clinical Genetics, Section Complex Trait Genetics, Amsterdam Neuroscience, Vrije Universiteit Medical Center, Amsterdam University Medical Centre, Amsterdam 1081 HZ, The Netherlands

**Keywords:** connectivity, GWAS, networks, neuroimaging, resting-state, structure-function

## Abstract

Functional connectivity within resting-state networks (RSN-FC) is vital for cognitive functioning. RSN-FC is heritable and partially translates to the anatomic architecture of white matter, but the genetic component of structural connections of RSNs (RSN-SC) and their potential genetic overlap with RSN-FC remain unknown. Here, we perform genome-wide association studies (*N*_discovery_ = 24,336; *N*_replication_ = 3412) and annotation on RSN-SC and RSN-FC. We identify genes for visual network-SC that are involved in axon guidance and synaptic functioning. Genetic variation in RSN-FC impacts biological processes relevant to brain disorders that previously were only phenotypically associated with RSN-FC alterations. Correlations of the genetic components of RSNs are mostly observed within the functional domain, whereas less overlap is observed within the structural domain and between the functional and structural domains. This study advances the understanding of the complex functional organization of the brain and its structural underpinnings from a genetics viewpoint.

## Significance Statement

Brain regions with synchronized activity can be clustered into distinct networks. We investigate which genetic effects contribute to structural (SC) and functional (FC) connectivity within seven networks and assess their degree of shared genetic signal. Multiple genetic effects are identified and highlight relevant biological processes for brain connectivity and brain disorders related to the networks. Overlap between the genetics of network connectivity is mostly observed within the functional domain. These results advance our biological understanding of the complex functional organization of the brain and its structural underpinnings, and their relevance for the genetics of neuropsychiatry.

## Introduction

Structural (SC) and functional connectivity (FC) are vital for healthy cognitive behavior (Buckholtz and Meyer-Lindenberg, 2012; de Lange et al., 2019). Brain regions that show temporally synchronized activity form functionally specialized resting-state networks (RSNs; Yeo et al., 2011), including primary networks (such as the visual or somatomotor network) and higher-order cognitive networks (such as the frontoparietal network, salience network, or default mode network; Bressler and Menon, 2010). Many psychiatric and neurologic disorders have been associated with disruptions within specific RSNs (van den Heuvel and Sporns, 2019). Improving our understanding of the biological principles underlying the concept of structural and functional connectivity within RSNs (RSN-SC/FC) could help elucidate the neural basis of human cognition and disorders associated with disruptions in brain connectivity.

Studies have shown that genetic factors significantly contribute to RSN functional connectivity (twin-based heritability *H^2^* = 20–40%; Meda et al., 2014; Ge et al., 2017; Adhikari et al., 2018; Miranda-Dominguez et al., 2018; Anderson et al., 2021; Barber et al., 2021). Genome-wide association studies (GWAS) on functional connectivity graph measures (Foo et al., 2021) and extrinsic and intrinsic functional organization of RSNs (Zhao et al., 2022) have identified the first genetic variants and sets of genes that make up this genetic component (heritability based on additive common genetic variants, mean $h_{SNP}^{2}$ = 13.3%). The genetic determinants of functional connectivity overlap with those of psychiatric disorders (Roelfs et al., 2022). Although RSNs were traditionally discovered based on functional correlation patterns between regions, structural connectivity properties of white matter between the respective brain regions correlate with functional connectivity (Batista-García-Ramó and Fernández-Verdecia, 2018; Mollink et al., 2019; van den Heuvel et al., 2009) to varying degrees across RSNs (Gu et al., 2021). The genetic architecture of RSN structural connectivity has not been investigated to date, but the substantial heritability of multiple properties of major white matter tracts (mean $h_{SNP}^{2}$ 25.18–34.9%; Smith et al., 2021; Zhao et al., 2021; Sha et al., 2023) suggests the importance of genetic factors for the anatomic backbone of RSNs. Describing the genetic architecture of both structural and functional RSN connectivity properties as well as annotation and interpretation of the genetic signal can give insight into a biological substrate relevant to a wide variety of neurologic and psychiatric disorders (de Lange et al., 2019) and additionally enables us to estimate to which degree structural and functional RSN connectivity properties are based on a shared genetic source.

This study is aimed to characterize the genetic architecture of within-RSN connectivity properties, both structurally and functionally. Large-scale (discovery *N*_functional_ = 24,336 and *N*_structural_ = 23,985; replication *N*_functional_ = 3408 and *N*_structural_ = 3412) GWAS are performed on the functional connectivity strength within seven well-known RSNs (Yeo et al., 2011) and a structural connectivity property (fractional anisotropy) within the same RSN definition. These 7 RSN are often used and providing GWAS summary statistics based on the same definition as most neuroimaging studies has the benefit of comparing genetic findings with phenotypic findings. A more granular definition (for example, 17 RSNs by Yeo et al., 2011) was not feasible within our univariate GWAS design, given that the accompanying multiple testing burden would drastically decrease our statistical power to identify and describe genome-wide significant loci (for a multivariate GWAS approach on 17 RSN, see Roelfs et al., 2022). With the polygenic signal from our GWAS, we estimate and partition the heritability and examine the convergence onto genes and biological pathways, with the purpose of aiding the biological interpretation of results and providing meaningful starting points for functional follow-up experiments (Uffelmann and Posthuma, 2020). We examine genetic correlations between different RSNs, as well as across structural and functional domains. These genetic correlation analyses are extended to the locus level to facilitate the prioritization of possible pleiotropic loci for future studies (Werme et al., 2022). Altogether, we focus on the translation of RSN-associated genetic loci into biological interpretation and provide insights into the genetic architecture of within-RSN functional and structural connectivity properties.

## Materials and Methods

A flowchart that describes all methods used in this manuscript is displayed in Extended Data Figure 1-3.

### Sample

The UK Biobank (UKB) is a resource with genomic and imaging data of volunteer participants (Sudlow et al., 2015). The National Research Ethics Service Committee North West–Haydock ethically approved this initiative (reference 11/NW/0382) and data were accessed under application #16406. Combined single nucleotide polymorphism (SNP)-genotypes and neuroimaging data of *N* = 40,682 participants have been available since January 2020. From all new subjects in the latest neuroimaging release (January 2020), we randomly assigned 5000 subjects to a holdout set for validation. Subsetting the total sample to subjects with all neuroimaging data necessary to construct our phenotypes as described below, resulted in *N*_functional_ = 37,017 and *N*_structural_ = 36,645. We only included subjects for which the projected ancestry principal component score was closest to and <6 SD from the average principal component score of the European 1000 Genomes sample based on Mahalanobis distance. This procedure has been described in previous publications by our group (Jansen et al., 2020) and the number of non-European exclusions are displayed in Extended Data Figure 1-2. Other exclusion criteria were withdrawn consent, UKB-provided relatedness, discordant sex, or sex aneuploidy (Extended Data Fig. 1-2). Further quality control on genomic and neuroimaging data are described below and resulted in the sample sizes and sample characteristics as displayed in Extended Data Figure 1-1.

### Genotype data

The genotype data used in this study for discovery and validation were obtained from the UK Biobank Axiom and the UK BiLEVE Axiom arrays. These Affymetrix arrays cover 812,428 unique genetic markers and overlap 95% in SNP content. This number of SNPs was increased to 92,693,895 by imputation conducted by UKB. Variants were imputed using the Haplotype Reference Consortium and the UK10K haplotype panel as reference. We applied our in-house quality control pipeline in addition to quality control performed by UKB. This procedure excluded SNPs with low imputation scores (INFO < 0.9), low minor allele frequency (MAF < 0.005) or high missingness (>0.05), multiallelic SNPs, indels, and SNPs without unique rs-identifiers. A total of 9,380,668 SNPs passed quality control and were converted to hard call SNPs using a certainty threshold of 0.9 for further analyses.

### Neuroimaging data

#### Preprocessing and connectome reconstruction

The UKB scanning protocol and processing pipeline is described in the UKB Brain Imaging Documentation (Smith, et al., 2020). For this study, we made use of the available resting-state functional brain images (rsfMRI) and multiband diffusion brain images (DWI) together with T1 surface model files and structural segmentation from FreeSurfer (Fischl, 2012). These three types of data were used as input for the structural and functional pipeline of CATO (Connectivity Analysis TOolbox; de Lange et al., 2022). Before this, UKB performed preprocessing on DWI and rsfMRI data as described in the UKB Brain Imaging Documentation (Smith et al., 2020).

In CATO’s structural pipeline, additional preprocessing of DWI files was performed in FSL (Jenkinson, et al., 2012) by computing a DWI reference image based on the corrected diffusion-unweighted (b0) volumes, computing the registration matrix between DWI reference image and the anatomic T1 image, and registering the Freesurfer segmentation to the DWI reference image. The surface was parcellated based on the Cammoun subparcellations of the Desikan–Killiany atlas including 250 cortical regions (Cammoun et al., 2012). We reconstructed the diffusion signal with diffusion tensor imaging (DTI), a deterministic method that is robust and relatively simple compared with more advanced diffusion reconstruction methods (de Lange et al., 2022). In CATO, the fiber assignment by continuous tracking (FACT) algorithm (Mori et al., 1999) is used to reconstruct fibers and fractional anisotropy (FA) was used as weights of reconstructed fibers. FA is a relatively simple measure of white matter integrity and correlates with axon density, size and myelination (Beaulieu, 2002). The structural connectivity matrix was built out of all fiber segments that connected two regions in the atlas. Additional filters were applied, namely a minimal FA of 0.1, minimal length of 30 mm and having 2 or more number of streamlines.

The functional pipeline in CATO consisted of similar steps. First, we computed an rsfMRI reference image by averaging all rsfMRI frames in FSL and subsequently registered this reference image and the T1 image in FreeSurfer. Second, we parcellated the surface based on the same atlas as in the structural pipeline (to enable structure-function comparison in downstream analyses) and we registered the T1 parcellation to the rsfMRI image. Third, motion metrics were estimated, and time-series were corrected for covariates (linear trends and first order drifts of motion parameters and the mean signal intensity of voxels in white matter and cerebrospinal fluid and of all voxels in the brain) by regression. Fourth, time-series were passed through bandpass filtering (frequencies 0.01–0.1) and scrubbing (max FD = 0.25, max DVARS = 1.5, min violations = 2, backward neighbors = 1, forward neighbors = 0). Fifth, the functional connectivity matrix was computed by the Pearson’s correlation coefficient of the average signal intensity of every pair of brain regions across the frames that survived filtering.

#### Quality control

The UKB scanning and preprocessing protocol includes filters for outliers based on manual QC and an advanced classifier described elsewhere (Alfaro-Almagro et al., 2018). We excluded a small number of subjects that UKB identified as outliers and placed in an “unusable” folder. The UKB main documentation (Smith et al., 2020) suggests a second set of UKB data fields that can be used as outlier criteria. Outlier subjects are defined as subjects that score for any of the values >3 interquartile ranges above the upper quartile or below the lower quartile. Outlier criteria included measures that describe the discrepancy between the T1-weighted, rsfMRI and DWI images and the population average template after LINEAR and NON-LINEAR alignment, the amount of nonlinear warping necessary to map a subject to the standard template, the signal-to-noise ratio in rsfMRI, the mean rfMRI head motion averaged across space and time points and the total number of outlier slices in DWI volumes. We extended this recommended list with connectome specific measures, including the average prevalence of all connections present and absent in the reconstructed brain network of a subject (low average prevalence scores indicate the presence of odd connections and high values indicate the absence of common connections), the sum of number-of-streamlines and average FA of all connections in the reconstructed brain network of a subject. The number of exclusions can be viewed in Extended Data Figure 1-2.

#### Phenotype reconstruction

In this study, the phenotypes of interest were functional and structural connectivity properties (FC; SC) within seven resting-state networks (RSNs) that previously have been identified (Yeo et al., 2011) and are commonly used in (clinical) neuroimaging studies: the default mode network, ventral attention network, dorsal attention network, visual network, limbic network, somatomotor network and frontoparietal network. Each of the 250 cortical regions of the reconstructed structural and functional connectomes were assigned the ratio to what extent they belonged to each of these seven RSNs, using a mask created and validated elsewhere (see Wei et al., 2019). Each connection was then weighted by multiplying the ratios of the two regions involved in the particular RSN. FC and SC properties within the RSNs were, respectively, calculated as the mean correlation and mean fractional anisotropy of the connections within the RSN. We also computed two global FC and SC phenotypes as the mean correlation and mean fractional anisotropy of all available connections, to be able to correct for connectivity that is nonspecific to RSNs in downstream analyses.

### Statistical analyses

#### SNP-based GWAS

To identify common genetic variants involved in FC within each of the seven RSN, we performed seven single nucleotide polymorphism (SNP)-based genome-wide association studies (GWAS) in PLINK2 (Purcell et al., 2007). Also, for the SC property within each of the seven RSN, a SNP-based GWAS was performed. It is common practice to include a global FC or SC estimate as covariate in GWAS to capture associations that are driven by the level of connectivity within an RSN regardless of the level of connectivity throughout the whole brain. It has become apparent that this risks the introduction of collider bias (inducing false-positives; Day et al., 2016). Here, we build on recent developments in statistical genetics that have provided multiple methods that allow for post-GWAS analyses conditional on global connectivity. Therefore, we used the global FC and global SC phenotypes to run two additional SNP-based GWAS, for which the summary statistics were used for conditional downstream analyses. The total number of GWASs was therefore 16. In order to correct for population stratification during GWAS, a principal component analysis was performed in FlashPCA2 (Abraham et al., 2017) using only independent (*r*^2^ < 0.1), common (MAF > 0.01), and genotyped SNPs or SNPs with very high imputation quality (INFO = 1). The first 30 principal components were used as covariates in all GWASs, together with sex, age, genotype array, Townsend deprivation index (a proxy of socio-economic status), and general neuroimaging confounders as well as FC/SC specific covariates (recommended by Alfaro-Almagro et al., 2020). The general set included handedness, scanning site, the use of T2 FLAIR in Freesurfer processing, intensity scaling of T1, intensity scaling of T2 FLAIR, scanner lateral (X), transverse (Y), and longitudinal (Z) brain position, and *z*-coordinate of the coil within the scanner. FC-specific and SC-specific covariates were, respectively, intensity scaling and echo time of rsfMRI, and intensity scaling of DWI. For reasons of collinearity, we ran principal component analysis on all covariates (excluding the population stratification principal components) and retained those principal components that explained > 99% of variance. Rare variants (MAF < 0.005) and SNPs with high missingness (>5%) were excluded from GWAS and male X variants were counted as 0/1. The genome-wide significance threshold was α = (0.05/1,000,000/16 =) 3.13 × 10^−9^ according to the Bonferroni correction for multiple testing.

#### SNP-based heritability

SNP-based ( $h_{SNP}^{2}$; or narrow-sense) heritability represents the proportion of phenotypic variance that can be explained by common additive variation. In contrast, broad-sense heritability captures the total genetic contribution to the phenotype and is often based on family studies (Visscher et al., 2008). We applied linkage disequilibrium score regression (LDSC) on the SNP-based GWAS summary statistics of all 16 phenotypes to estimate $h_{SNP}^{2}$ using precomputed LD scores from 1000 Genomes EUR, as provided by the LDSC developers.

For the phenotypes with enough polygenic signal to run LDSC (λ > 1.02), we investigated whether certain functional categories in the human genome were enriched for $h_{SNP}^{2}$. The ratio of the proportion of $h_{SNP}^{2}$ in a certain category to the proportion of SNPs in the category equaled the enrichment value. We corrected the level of significance for multiple testing to α = (0.05/28)/11 = 1.62 × 10^−4^.

#### Functional annotation

Functional mapping and annotation (FUMA) is a web-based platform that can be used to functionally map and annotate SNPs that appear significant during GWAS. We uploaded summary statistics to FUMA if GWAS identified at least one genome-wide significant SNP. Candidate SNPs were defined as all SNPs in linkage disequilibrium (LD) *r*^2^ > 0.6 with an independent genome-wide significant SNPs (*r*^2^ < 0.6). Annotation was subsequently performed using ANNOVAR (K. Wang et al., 2010), RegulomeDB score (Boyle et al., 2012) and ChromHMM (Ernst and Kellis, 2012). Lead SNPs were defined as independent SNPs *r*^2^ < 0.1. Genomic loci were constructed by taking all independent significant SNPs *r*^2^ < 0.1 with LD blocks within 250 kb distance and independent significant SNPs *r*^2^ ≥ 0.1. Within every locus, SNPs were mapped to genes using three methods: positional mapping, expression quantitative trait loci (eQTL) mapping or chromatin interaction mapping. SNPs were positionally mapped to genes if their physical distance was <10 kb. Mapping based on eQTLs relied on known associations between SNPs and the gene expression of genes within a 1-Mb window, from BRAINEAC frontal, occipital, temporal and cerebral cortex (Ramasamy et al., 2014), GTEx v8 cerebral cortex (GTEx Consortium, 2020) and xQTLServer dorsolateral prefrontal cortex (Ng et al., 2017). Chromatin interaction mapping was based on established 3D DNA-DNA interactions between SNP and gene regions from Hi-C databases in cortex tissue [PsychENCODE by D. Wang et al. (2018), Giusti-Rodriguez et al. (2019) and GSE87112 (Schmitt et al., 2016)]. To restrict chromatin interaction mapping to plausible biological interactions, we only included interactions where one region overlapped with an enhancer [as predicted by the Roadmap Epigenomics Consortium (2015) in cortex tissue] and the other region overlapped with a promoter (250 bp upstream to 500 bp downstream of the transcription start site as well as predicted by the Roadmap Epigenomics project in cortex tissue). A false discovery rate (FDR) threshold of 1 × 10^−5^ was used, as recommended in previous literature (Schmitt et al., 2016).

#### Statistical fine-mapping of genome-wide significant loci

Statistical fine-mapping methods can aid in determining the probability of variants identified through GWAS being causal. We applied FINEMAP to the genome-wide significant loci defined by FUMA, which is a Bayesian statistical fine-mapping tool that estimates the posterior probability of a specific model by combining the prior probability and the likelihood of the observed summary statistics (Benner et al., 2016). The posterior probabilities are used to calculate the posterior inclusion probability (PIP) of a SNP in a model and the minimum set of SNPs needed to capture the SNPs that most likely cause the association (Schaid et al., 2018). We set the maximum number of causal SNPs to *k *=* *10 and report on the 95% credible set of the model with *k* causal SNPs reaching the highest probability. LDstore (Benner et al., 2017) was used to estimate the pairwise LD matrix of SNPs from quality controlled genomic data of the discovery sample. Only SNPs with PIP > 0.10 were used for interpretation in the main text, but all are reported on in Extended Data Figure 3-2.

#### Gene-based GWAS

Performing GWAS on the level of genes has been suggested to be more powerful than GWAS on the level of SNPs (de Leeuw et al., 2015). Therefore, the 16 SNP-based GWAS summary statistics were used to perform 16 gene-based GWAS in MAGMA (multimarker analysis of genomic annotation) v1.08 (de Leeuw et al., 2015). A mean SNP-wise model was applied (with the UKB European population serving as an ancestry reference group) to test the joint association of all SNPs within 18,850 genes with FC/SC within RSNs. The genome-wide significance threshold was adjusted for multiple testing to α = (0.05/18,850)/16 = 1.66 × 10^−7^.

#### Gene-set analysis

We set out to prioritize the associations from gene-based GWAS and gain more insight into the biological pathways associated to RSN-FC/RSN-SC. In order to identify gene-sets specific for RSN-FC/RSN-SC, we ran conditional gene-set analyses in MAGMA conditioning on the global FC or SC, respectively. Pathways were represented as gene-sets from Gene Ontology (GO) molecular functions, cellular components and biological processes, and curated gene-sets from MsigDB v7.0 (sets C2 and C5; Liberzon et al., 2011). Protein-coding genes served as background genes. The threshold for gene-sets reaching significance was corrected for multiple testing to α = (0.05/7246)/14 = 4.93 × 10^−7^.

#### Genome-wide genetic correlations

**Figure 1.**
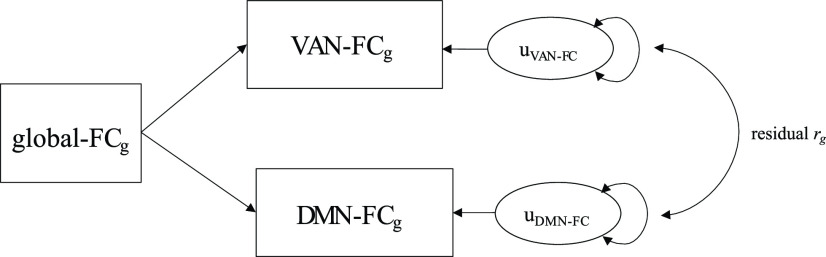
Path diagram of genomic SEM model. The summary statistics of two RSNs that have shown to significantly correlate with global connectivity will be used as input together with summary statistics of the global connectivity GWAS. In this way, *r*_g_ between the unique components (u) of the two RSNs can be estimated while taking global connectivity into account. The example in this diagram shows the global and unique genetic effects on functional connectivity (FC) for the ventral attention network (VAN) and default mode network (DMN), but a similar model was used for other RSN pairs and for measures of structural connectivity (SC). This method is embedded in a flowchart that describes the sample (see Extended Data Figs. 1-1 and 1-2 for sample characteristics and exclusion criteria) and all methods used in this manuscript (Extended Data Fig. 1-3).

We aimed to test whether genetic signals were generally similar across the FC/SC within various RSNs. To assess the overlap in genetic architecture between FC/SC within RSNs while taking the influence of global FC/SC into account, we designed a genetic correlation (*r*_g_) analysis pipeline. This pipeline consisted of three steps. (1) In the first step, genome-wide *r*_g_ between 42 combinations of RSNs were estimated using LDSC [α = (0.05/42=) 1.19 × 10^−3^]. The summary statistics of SNP-based GWAS were used as input for LDSC. We excluded FC within the visual network, because both the λ (<1.02) and ratio (>0.20) values were out of bound for LDSC. (2) For all RSNs included in a significant bivariate *r*_g_, additional *r*_g_ with global FC/SC were calculated in LDSC. (3) If one or both RSNs from the significant bivariate *r*_g_ showed additional significant *r*_g_ with global FC/SC, we recalculated of the genome-wide *r*_g_ between the two RSNs with global FC/SC taken into account. Since such residual genome-wide *r*_g_ analyses are not implemented in LDSC, we applied genomic structural equation modelling (genomic SEM; Grotzinger et al., 2019). Genomic SEM is a method that enables to model the multivariate genetic architecture and covariance structure of complex traits using GWAS summary statistics and allows for sample overlap. We modelled residual covariance between RSNs as the covariance between the residual variance of the two RSNs involved after taking the global factor into account (Fig. 1). The model was then fit using diagonally weighted least square estimation.

10.1523/ENEURO.0242-22.2023.f1-1 Extended Data Figure 1-1 Mean age (years) and female percentage in discovery and holdout samples for GWAS on RSN-FC/RSN-SC. Download Figure 1-1, XLS file.

**Figure 2.**
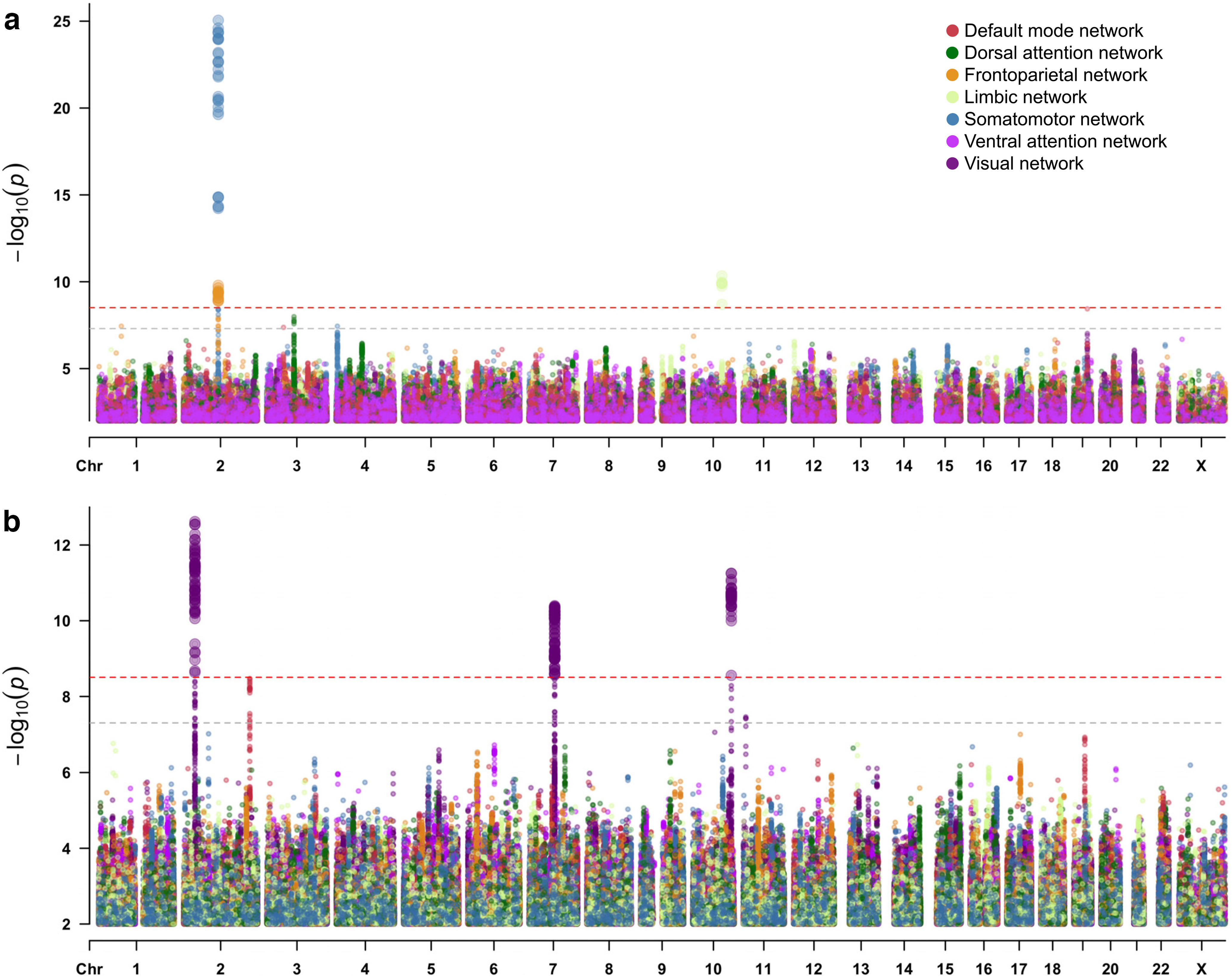
Multitrait Manhattan plots of SNP-based GWAS for (***a***) within-RSN functional connectivity strength (RSN-FC) and (***b***) within-RSN structural connectivity property (RSN-SC). The light gray dashed horizontal line indicates traditional genome-wide significance (*p *<* *5 × 10^−8^), whereas the red dashed horizontal line indicates genome-wide significance after additional correction for the number of traits tested (*p *<* *3.13 × 10^−9^). SNPs with *p *>* *0.01 are omitted for visualization purposes. Manhattan plots per RSN are provided as Extended Data Figures 2-1 (FC) and 2–2 (SC), replication efforts of these results (Extended Data Figs. 2-3, 2-4) are plotted in Extended Data Figure 2-5, and characteristics of all loci, lead, and candidate SNPs are available in Extended Data Figures 2-6 and 2-7. Heritability estimates based on these GWAS results are provided in Extended Data Figures 2-8 and 2-9.

10.1523/ENEURO.0242-22.2023.f1-2 Extended Data Figure 1-2 Sample size and exclusion criteria for discovery and holdout sample in GWAS for RSN-FC/RSN-SC. From all subjects in the latest neuroimaging release, we randomly assigned 5000 subjects to a holdout set for validation. Genotype quality control exclusion criteria included UKB-provided relatedness, discordant sex or sex aneuploidy. Outliers include “ununsable” subjects as well as outliers based on methods advised by UKB Neuroimaging Documentation. Download Figure 1-2, XLS file.

**Figure 3.**
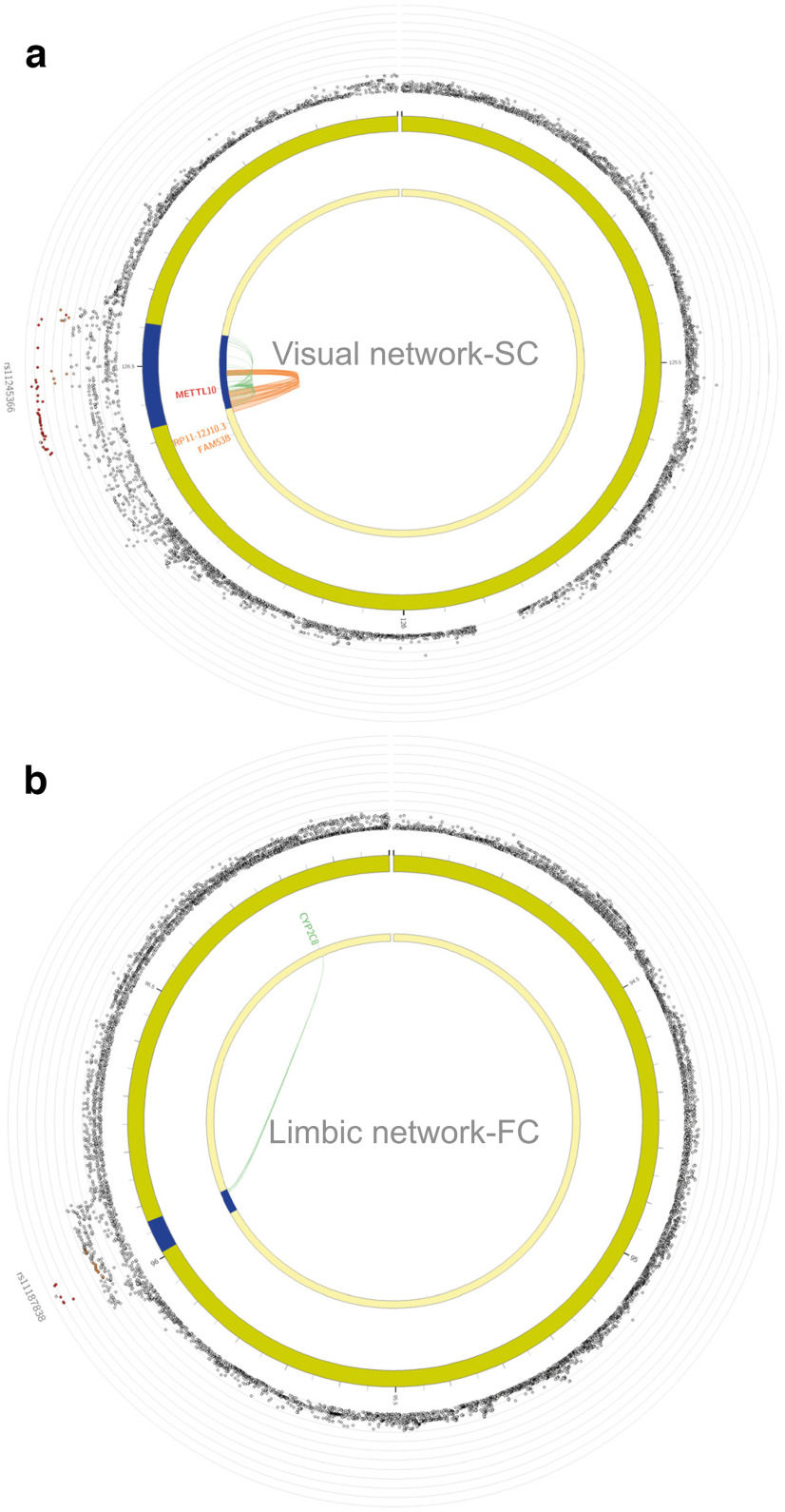
eQTL and Hi-C gene mapping of structural connectivity (SC) and functional connectivity (FC) network measures. ***a***, Within-visual network-SC SNPs were mapped to METTL10, FAM53B, and METTL10-FAM53B readthrough (RP11-12J10.3) through chromatin interaction mapping (orange). METTL10 was additionally mapped by 46 SNPs because of their eQTL associations in cerebral cortex tissue. ***b***, FUMA gene mapping, based on established eQTL associations (green) in human temporal cortex, link eight within-limbic network-FC SNPs on chromosome 10 to CYP2C8. All FUMA gene-mapping results are displayed in Extended Data Figure 3-1, with fine-mapping results in Extended Data Figure 3-2.

10.1523/ENEURO.0242-22.2023.f1-3 Extended Data Figure 1-3 Flowchart of methods involved in the current study. Functional and structural connectivity (FC/SC) within resting-state networks (RSN) as defined by Yeo et al. (2011) were obtained similarly as previously described (Wei et al., 2019). SNP-based and gene-based GWAS and in silico follow-up were performed on a discovery sample and were validated in a replication sample. rsfMRI = resting-state functional magnetic resonance imaging, DWI = diffusion weighted imaging, SNP = single nucleotide polymorphism, FC = functional connectivity, SC = structural connectivity, GWAS = genome-wide association study, LDSC = linkage disequilibrium score regression, LAVA = local analysis of [co]variant annotation, FUMA = functional mapping and annotation, MAGMA = multimarker analysis of genomic annotation, MsigDB = molecular signatures database, PGS = polygenic score. Download Figure 1-3, TIF file.

10.1523/ENEURO.0242-22.2023.tab1-1 Extended Data Table 1-1 Bivariate and partial local genetic correlations from LAVA between summary statistics of RSN-FC/RSN-SC. Locus = locus number. chr = chromosome of locus. start = basepair start position of the locus. stop = basepair end position of the locus. n.snps = number of snps within the locus. n.pcs = number of PCs within the locus. phen1 = one of the two phenotypes involved in the bivariate *r*_g_. phen2 = one of the two phenotypes involved in the bivariate *r*_g_. z = the conditional phenotype in partial *r*_g_, rho = local genetic correlation. rho.lower and rho.upper = 95% confidence interval of rho. r2.phen1 z and r2.phen2 z = the proportion of genetic variance explained for phen1/phen2 by z. pcor = partial correlation coefficient. ci.lower and ci.lower = 95% confidence interval for partial correlation coefficient; P = *p*-value for local *r*_g_. Download Table 1-1, XLS file.

#### Local genetic correlations

The genome-wide *r*_g'_s described above are an average correlation of genetic effects across the genome, implicating that contrasting local *r*_g’_s are possibly cancelling each other out. Running *r*_g_ analysis on a locus level has the potential to uncover loci that show genetic similarity between traits. For this purpose, we adopted a three-step local *r*_g_ analysis pipeline similar to the genome-wide *r*_g_ analysis approach described above. All three steps were performed in LAVA (Werme et al., 2022), a local *r*_g_ analysis *R* package, using SNP-based GWAS summary statistics as input. We followed the suggested sample overlap procedure (as described on https://github.com/josefin-werme/LAVA) to enable LAVA to model shared variance because of sample overlap as residual covariance and consequently remove upward bias in local *r*_g_ estimates (Werme et al., 2022). Since our GWASs included European samples, the 1000 Genomes Phase 3 European data served as genotype reference and formed the basis of the locus definition file. For every locus, the first step of our pipeline consisted of estimating local bivariate *r*_g_ between 49 combinations of RSNs. However, RSNs that were devoid of heritable signal (*p *>* *1 × 10^−4^) in the locus were excluded from local bivariate *r*_g_ analysis to ensure interpretability and reliability. A total of 774 bivariate tests were performed across 337 loci, leading to an adjusted significance threshold of α  = (0.05/774=) 6.46 × 10^−5^. In the second step, RSNs that showed significant local *r*_g_ were additionally tested for *r*_g_ in that locus with global FC/SC. Note that if this was not possible, because global FC/SC showed no significant heritability in that locus, the local bivariate *r*_g_ between RSNs could not be biased by global FC/SC. If one or both RSNs did show additional significant *r*_g_ with global FC/SC, we ran a partial local *r*_g_ between the RSNs conditioned on the SC-global and/or FC-global phenotype in step three. If the partial local *r*_g_ between the RSNs no longer remained significant, we concluded that the initial *r*_g_ was driven by global FC/SC and did not reflect genetic overlap specific for these RSNs.

#### Polygenic score

The variance explained in RSN-FC/RSN-SC by our GWAS findings was investigated to test the robustness of our findings using polygenic score (PGS) estimation in PRSice-2 (Choi et al., 2019). We applied a two-phase approach to obtain *p*-values unaffected by overfitting and therefore split our holdout sample in a target (*N* = 1818) and validation set (*N* = 1824). In phase 1, SNP-based summary statistics (MAF > 0.1, chromosome X excluded) from the discovery sample together with genotype data of the target set were used to find the optimal *p*-value threshold. PRSice-2 uses high-resolution thresholding and clumping of genotype data, and we included the same covariates as during discovery GWAS. In phase 2, the model with the best fit from phase 1 was applied on the validation set to obtain the variance explained (*R*^2^).

#### Replication of lead SNPs

In the design of this study, a hold-out sample of *N*_FC_ = 3408 and *N*_SC_ = 3412 was reserved for PGS analysis. We applied an earlier described method (see Okbay et al., 2016) to internally validate our discovery lead SNPs. This formula describes the probability of a discovery lead SNP *i* being significant in a replication sample as

$$P\left(sig_{i}\right)=\text{Φ}\left(-\frac{\left|\beta_{i}\right|}{\sigma_{rep,i}}+{\text{Φ}}^{-1}\left(\frac{\alpha }{2}\right)\right)+\left[1-\text{Φ}\left(-\frac{\left|\beta_{i}\right|}{\sigma_{rep,i}}-{\text{Φ}}^{-1}\left(\frac{\alpha }{2}\right)\right)\right],$$

with α representing an α level of 0.05, Φ the cumulative normal distribution function, ${\text{Φ}}^{-1}$ the inverse normal distribution function, $\sigma_{rep,i}$ the standard error of SNP *i* in the replication GWAS and $\beta_{i}$ the winner’s curse adjusted association estimate of SNP *i*. Winner’s curse is the occurrence of overestimated effect sizes that are induced by significance thresholding (Palmer and Pe’er, 2017). We applied winner’s curse correction using the mean of the normalized conditional likelihood (Ghosh et al., 2008) in the *winnerscurse* R package. The number of SNPs that is expected to show significance was then summed across all six lead SNPs by $\underset{i}{\sum }P(sig_{i})$. Given the small effect sizes of GWAS SNPs, a larger sample size is often needed to replicate findings. Since the standard error of a SNP is dependent on sample size, we calculated $P\left(sig_{i}\right)$ for a range of sample sizes by $\frac{SD_{rep,i}}{\sqrt{N}}$ and plotted $P\left(sig_{i}\right)$ across this range to describe the power to replicate these lead SNPs.

#### Code availability

No new software was developed for this project, existing software and code are publicly available: CATO, http://www.dutchconnectomelab.nl/CATO/; FUMA, http://fuma.ctglab.nl/; MAGMA, https://ctg.cncr.nl/software/magma; LDSC, https://github.com/bulik/ldsc; PRSice-2, https://www.prsice.info; LAVA, https://github.com/josefin-werme/LAVA; genomic SEM, https://github.com/GenomicSEM/GenomicSEM; PLINK, https://www.cog-genomics.org/plink/; FLASHPCA, https://github.com/gabraham/flashpca; winnerscurse R package, https://amandaforde.github.io/winnerscurse/.

#### Data availability

Genome-wide summary statistics will be made publicly available via https://ctg.cncr.nl/software/summary_statistics/ upon publication.

## Results

### GWAS of RSN-SC and RSN-FC properties identify six genome-wide significant loci

Following previously described procedures (Wei et al., 2019), we started our analysis by grouping cortical areas into seven RSN as defined by Yeo et al. (2011; visual, somatomotor, limbic, dorsal attention, ventral attention, frontoparietal, and default-mode network; Extended Data Fig. 1-3) and calculating the mean functional and structural connectivity within the RSNs in UK Biobank subjects (discovery *N*_FC_ = 24,336 and *N*_SC_ = 23,985; replication *N*_FC_ = 3408 and *N*_SC_ = 3412). Within-RSN functional connectivity strength (from now on referred to as RSN-FC) was measured as the average correlation between the activation signals of brain regions within each RSN over time. A property of within-RSN structural connections (from now on RSN-SC) was measured as the average fractional anisotropy (FA) of white matter connections between brain regions within each RSN (see Materials and Methods). FA values are believed to reflect a metric of efficiency of or capacity for information transfer across white matter pathways and are sensitive to myelin content (Beaulieu, 2002). Discovery GWAS were performed for the FC and SC within every RSN and identified 518 genome-wide significant variants (or single nucleotide polymorphisms; SNPs) at *p* < (5 × 10^−8^/16 =) 3.13 × 10^−9^ located in six genomic loci (Fig. 2): three for visual network-SC, one for limbic network-FC, and a shared locus for frontoparietal network-FC and somatomotor network-FC (Extended Data Fig. 2-6). These loci do not seem simply driven by overall connectivity properties, given that none of these six loci showed a genome-wide significant association with global FC or SC.

10.1523/ENEURO.0242-22.2023.f2-1 Extended Data Figure 2-1 Manhattan plots of SNP-based (left) and gene-based (right) GWAS for RSN-FC. The light grey dashed horizontal line indicates (left) traditional GWS (*p *<* *5 × 10^−8^) or (right) significance after correcting for the number of genes tested per trait (*p *<* *2.65 × 10^−6^). The red dashed horizontal line indicates significance after an additional correction for the number of traits tested (left; *p *<* *3.13 × 10^−9^) or (right; *p *<* *1.66 × 10^−7^). Download Figure 2-1, EPS file.

10.1523/ENEURO.0242-22.2023.f2-2 Extended Data Figure 2-2 Manhattan plots of (left) SNP-based and (right) gene-based GWAS for RSN-SC. The light grey dashed horizontal line indicates (left) traditional GWS (*p *<* *5 × 10^−8^) or (right) significance after correcting for the number of genes tested per trait (*p *<* *2.65 × 10^−6^). The red dashed horizontal line indicates significance after an additional correction for the number of traits tested (left; *p *<* *3.13 × 10^−9^) or (right; *p *<* *1.66 × 10^−7^). Download Figure 2-2, EPS file.

10.1523/ENEURO.0242-22.2023.f2-3 Extended Data Figure 2-3 Best *p*-value threshold and PGS prediction (with PRSice) for FC/SC within RSNs. Phase 1 = GWAS of FC/SC within RSNs in discovery sample. Phase 2 = best *p*-value threshold determination in half replication sample. Phase 3 = validation of best *p*-value threshold PRS in other half replication sample. Threshold = best *p*-value threshold. PRS R2 = variance explained by the PRS. Full R2 = variance explained by the full model (including the covariates). Null R2 = variance explained by the covariates. Coefficient = regression coefficient of the model. SE = SE of regression coefficient of the model. P = *p*-value of the model fit. *N* SNPs = number of SNPs included in the model. Download Figure 2-3, XLS file.

**Figure 4.**
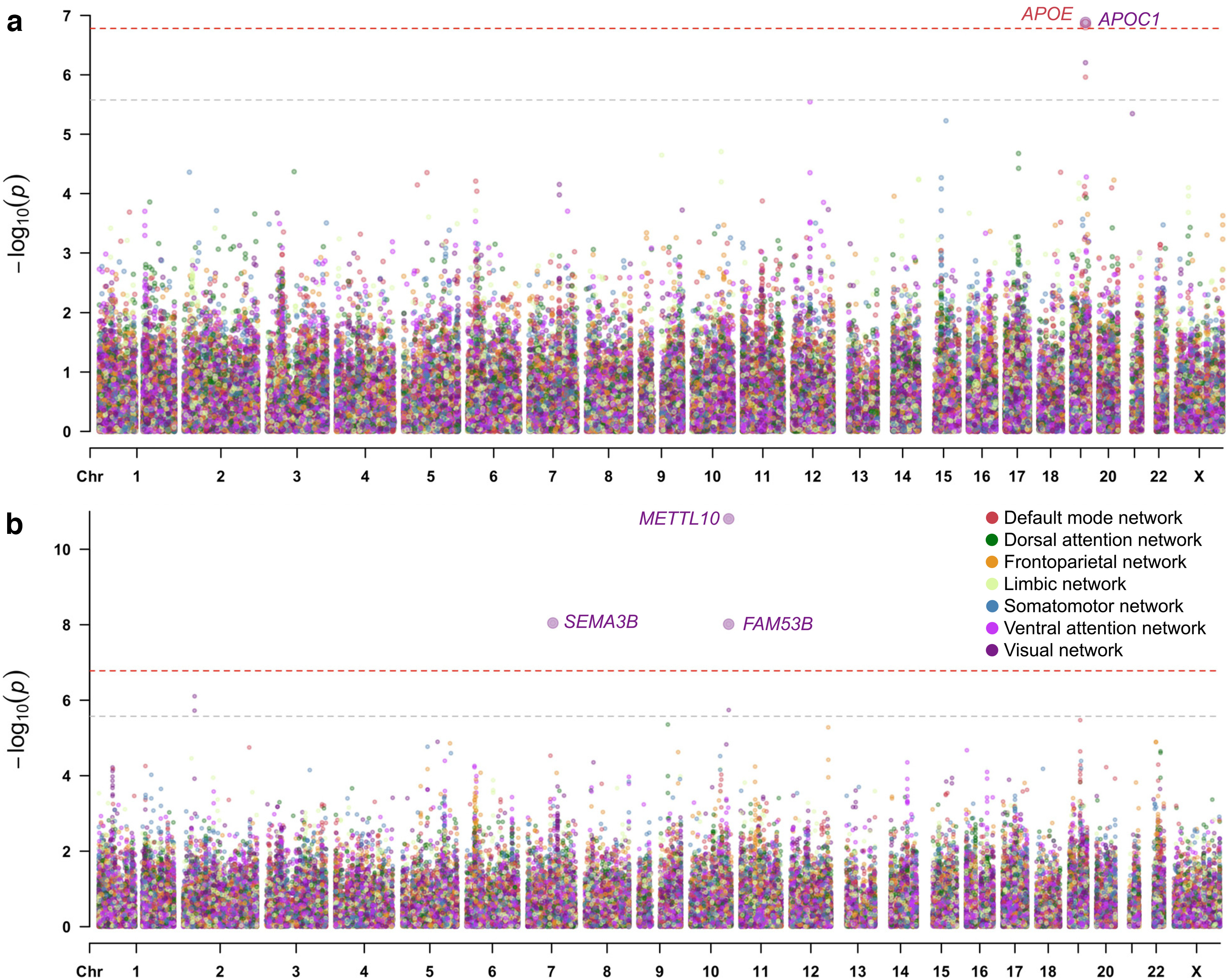
Multitrait Manhattan plots of gene-based GWAS for (***a***) FC and (***b***) SC within resting-state networks (RSNs). The light gray dashed horizontal line indicates significance after correcting for the number of genes tested per trait (*p* < 2.65 × 10^–6^), whereas the red dashed horizontal line indicates significance after an additional correction for the number of traits tested (*p* < 1.66 × 10^–7^). See Extended Data Figure 4-1 for the association *p*-values of all genome-wide significant genes, Extended Data Figure 4-2 for local *r*_g_ summary statistics between Alzheimer’s disease and default mode network-FC (plotted in Extended Data Fig. 4-3), and Extended Data Figure 4-4 for gene-set analysis statistics.

10.1523/ENEURO.0242-22.2023.f2-4 Extended Data Figure 2-4 Association statistics for discovery lead SNPs in the holdout set for replication. Phenotype = phenotype of the discovery lead SNP. lead SNP = original UKB variant name. CHR = chromosome. POS = position. REF = reference allele. ALT = alternative allele. A1 = effect allele. OBS_CT = per SNP sample size. BETA = effect size. SE = SE of the effect size. L95 = the lower endpoint of the confidence interval. U95 = the upper endpoint of the confidence interval. TSTAT = *t* statistic. P = *p*-value. ALT_FREQS = frequency of alternative allele. RSID_UKB = rs identifier of the SNP. MAF_UKB = MAF as provided by UKB (full file). INFO_UKB = INFO as provided by UKB (full file). Download Figure 2-4, XLS file.

**Figure 5.**
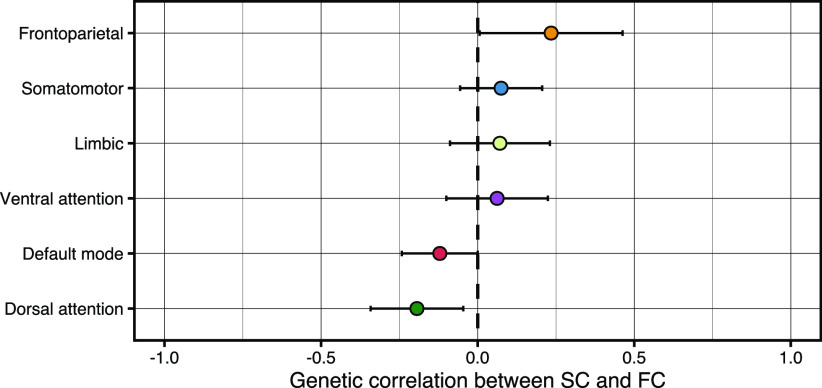
Global *r*_g_ (±SE) between functional connectivity (FC) and structural connectivity (SC) within the same RSN. Genetic correlations as performed in LDSC do not show estimates significantly different from 0 (Extended Data Fig. 5-1). Additional estimation of local *r*_g_ did not yield significant overlapping loci between SC and FC within each RSN either. The colors correspond to the RSN colors in Figure 2 and 4.

10.1523/ENEURO.0242-22.2023.f2-5 Extended Data Figure 2-5 Probability curves of discovery lead SNPs being significant in a replication sample of increasing size [log10(N) scale]. The current replication sample size of this study is represented by the vertical grey line. FC, functional connectivity; SC, structural connectivity; SMN, somatomotor network; LN, limbic network; FPN, frontoparietal network; VN, visual network. Download Figure 2-5, EPS file.

**Figure 6.**
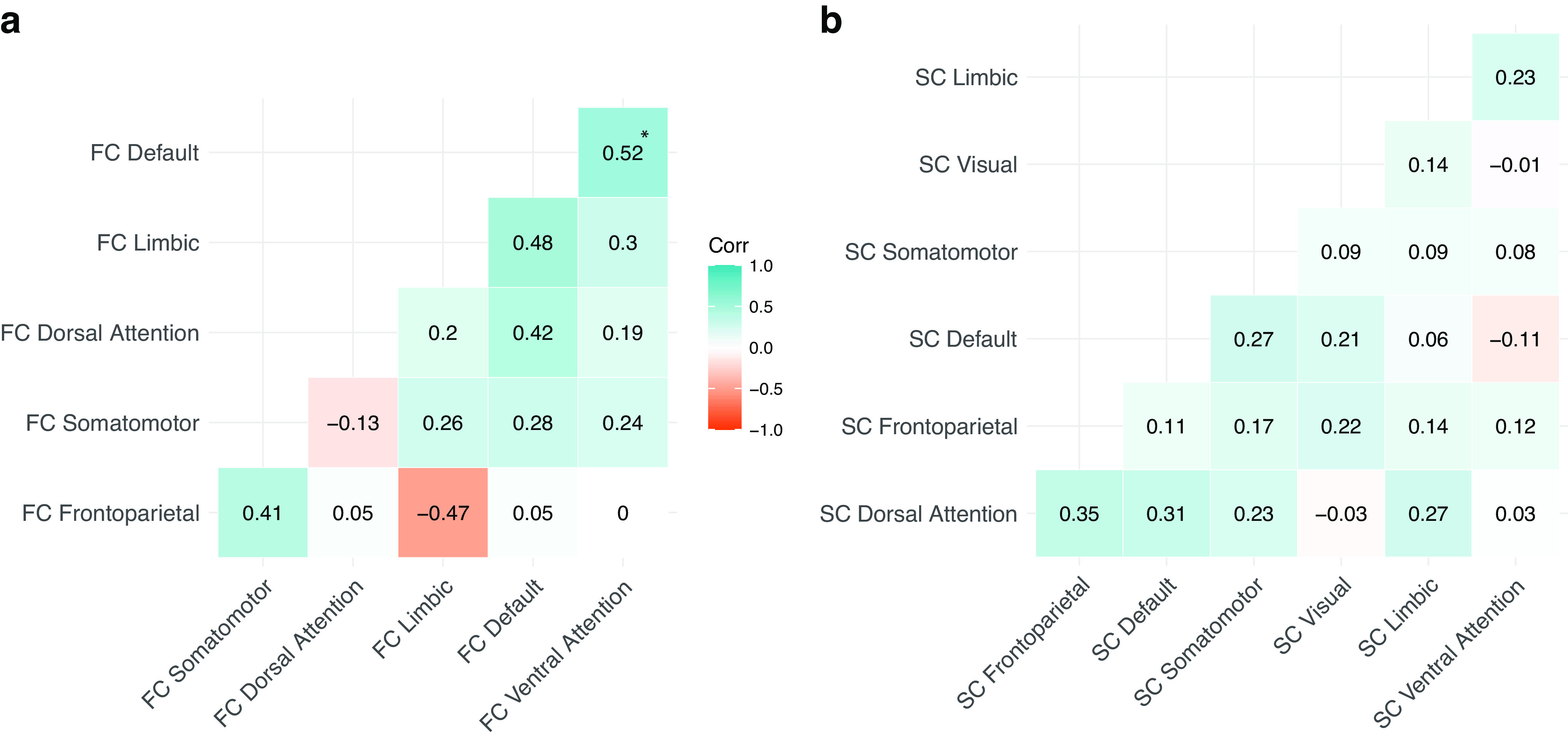
Genome-wide *r*_g_ across RSN measures of (***a***) functional connectivity (FC) and (***b***) structural connectivity (SC). If one of the two RSNs showing significant LDSC *r*_g_ showed additional significant *r*_g_ with global FC/SC, we instead report the residual *r*_g_ (*r*_g_ between the two RSNs while taking global FC/SC into account in Genomic SEM; see Materials and Methods and Fig. 1). The significant *r*_g_ that survived correction for multiple testing (*p *<* *1.19 × 10^−3^) is indicated with an asterisk (*).

10.1523/ENEURO.0242-22.2023.f2-6 Extended Data Figure 2-6 Genomic loci and lead variants in the GWAS of RSN-FC/RSN-SC as identified by FUMA. uniqID = ID of the top lead variant within the locus formatted as 'chromosome:base pair position:alleles in alphabetic order'. Chr = chromosome. Pos = base pair position lead SNP. P = *p*-value. Start = locus BP start position of the locus. End = stop position of the locus. nSNPs = number of unique candidate SNPs in the genomic locus, including non-GWAS-tagged SNPs (which are available in the use selected reference panel). nGWASSNPs = number of the GWAS-tagged candidate SNPs within the genomic locus. This is a subset of “nSNPs.” nIndSigSNPs = number of the independent significant SNPs in the genomic locus. IndSigSNPs = rsID of independent significant SNPs in the genomic locus. nLeadSNPs = the number of lead SNPs in the genomic locus. LeadSNPs = rsID of lead SNPs in the genomic locus. Download Figure 2-6, XLS file.

10.1523/ENEURO.0242-22.2023.f2-7 Extended Data Figure 2-7 FUMA output for all candidate SNPs. uniqID = unique ID of SNPs consists of chr:position:allele1:allele2 where alleles are alphabetically ordered. rsID = rsID of SNPs as provided in the input GWAS, otherwise extracted from the specified reference panel. chr = chromosome. pos = position on hg19. MAF = minor allele frequency computed based on the reference panel. P = *p*-value from cerebellar volume GWAS [non-GWAS tagged SNPs (extracted from the reference panel) are “NA”]. Beta = beta from cerebellar volume GWAS [non-GWAS tagged SNPs (extracted from the reference panel) are “NA”]. SE = SE of beta from cerebellar volume GWAS [non-GWAS tagged SNPs (extracted from the reference panel) are ““NA””). r2 = the maximum r2 of the SNP with one of the independent significant SNP. IndSigSNP = rsID of an independent significant SNP which has the maximum r2 of the SNP. Nearest gene = the nearest gene of the SNP based on ANNOVAR annotations. Dist = distance to the nearest gene. SNPs which are locating in the gene body or 1 kb upstream or downstream of TSS or TES have 0. Func = functional consequence of the SNP on the gene obtained from ANNOVAR. CADD = combined annotation dependent depletion score. RDB = RegulomeDB score. posMapFilt = whether the SNP was used for positional mapping or not, 1 is used, otherwise 0. eqtlMapFilt = whether the SNP was used for eQTL mapping or not, 1 is used, otherwise 0. ciMapFilt = whether the SNP was used for chromatin interaction mapping or not, 1 is used, otherwise 0. Download Figure 2-7, XLS file.

10.1523/ENEURO.0242-22.2023.f2-8 Extended Data Figure 2-8 SNP-based heritability of RSN-FC/RSN-SC. Heritability estimates were calculated using LD score regression (LDSC). h2 = heritability estimate. h2 SE = SE of heritability estimate. Intercept = the LDSC intercept (should be close to 1). Lambda = median(chi2)/0.4549. Mean chi2 = mean chi-square statistic. Ratio = (intercept-1)/(mean(chi2)-1), measures the proportion of the inflation in the mean chi^2 that the intercept ascribes to causes other than polygenic heritability. Ratio SE = SE of ratio. Download Figure 2-8, XLS file.

10.1523/ENEURO.0242-22.2023.f2-9 Extended Data Figure 2-9 Partitioned heritability of RSN-FC/RSN-SC in genomic categories. Partitioned heritability estimates were calculated using stratified LDSC regression. Prop. SNPs = proportion of total SNPs that belong to a given category (categories are not mutually exclusive; i.e., a SNP may be assigned to more than one category). Prop. h2 = proportion of heritability ascribed to SNPs in the category. Enrichment = proportion of heritability divided by proportion SNPs. Enrichment SE = SE of the enrichment estimate. Enrichment *p*-value = *p*-value of the enrichment estimate. Download Figure 2-9, XLS file.

The proportions of phenotypic variance explained by additive genetic effects of GWAS SNPs (SNP-based heritability; $h_{SNP}^{2}$) were moderately higher for RSN-SC (M = 13.59%, SD = 1.79%) than those observed for RSN-FC (M = 6.71%, SD = 3.36%; Extended Data Fig. 2-8). We did not find evidence for enrichments of $h_{SNP}^{2}$ in functional genomic categories after Bonferroni-correction (Extended Data Fig. 2-9). The linkage disequilibrium score regression (LDSC) intercept approached one for all phenotypes, indicating limited bias from population stratification.

### Axon guidance and synaptic functioning genes implicated in visual network-SC GWAS

We continued by examining the possible functional consequences of the SNPs involved in RSN-FC and RSN-SC. SNPs in linkage disequilibrium (i.e., in correlation; LD; *r*^2^ ≥ 0.6) with the Bonferroni-corrected genome-wide significant SNPs from the GWAS which also had suggestive *p*-values (<1 × 10^−5^) and a minor allele frequency (MAF) > 0.005 were defined as candidate SNPs (Extended Data Fig. 2-7). We subsequently mapped candidate SNPs to genes using three strategies (Materials and Methods): positional mapping if the SNP was within <10 kb of a gene, expression quantitative trait loci (eQTL) mapping if the SNP was known to affect expression of the gene within 1 Mb in cortex, and chromatin interaction mapping if there was a significant Hi-C interaction between the SNP and a nearby or distant gene in cortex tissue (Extended Data Fig. 3-1).

For visual network-SC, an exonic nonsynonymous SNP located in exon 1 of *AC007382.1* (rs711244, *p *=* *1.42 × 10^−12^) was among the candidate SNPs in the locus on chromosome 2. The function of *AC007382.1* is unknown, but it has been associated with amygdala volume previously (Mufford et al., 2021). A SNP 5 kb from *AC007382.1* (rs35050623) was also among the most likely causal SNPs as identified by fine-mapping the locus (posterior inclusion probability; PIP = 0.12). The loci on chromosome 10 and 7 were “unsolved” by FINEMAP (all PIPs < 0.1), which can occur because of a combination of extensive linkage disequilibrium and small effect sizes (Extended Data Fig. 3-2). Exonic synonymous SNPs were found in exon 7 of *FAM175B* and exon 12 of *SEMA3A* in the loci on chromosome 10 and 7, respectively. The transcript of *FAM175B* is a component of an enzyme complex that deubiquitinates Lys-63 linked chains to control protein function (Cooper et al., 2010). Experimental studies have suggested that such deubiquitination can regulate synaptic transmission and synaptic plasticity (DiAntonio and Hicke, 2004). *SEMA3A* contained multiple intronic SNPs associated with visual network-SC with high combined annotation dependent depletion (CADD) scores (11 SNPs with CADD > 12.37), which are usually considered reducing organismal fitness and correlating with molecular functionality and pathogenicity (Kircher et al., 2014). The product of *SEMA3A* is known as a key regulator of axon outgrowth during the establishment of correct pathways in the developing nervous system (Zhou et al., 2008).

We additionally mapped 46 visual network-SC candidate SNPs to *METTL10*, because of their established associations with *METTL10* expression levels in fetal and adult cerebral cortex tissue (eQTL mapping) as well as their chromatin interaction. *METTL10* encodes a methyltransferase that catalyses the trimethylation of eukaryotic elongation factor α (eEF1A) at Lys-318, a key regulator of ribosomal translation (Jakobsson et al., 2018). Visual network-SC SNPs were also mapped to the *METTL10-FAM53B* readthrough (*RP11-12J10.3*) and *FAM53B* gene, because of known chromatin interaction in fetal and adult cerebral cortex tissue (Fig. 3*a*). FAM53B is required for Wnt signaling, a pathway important for cell regeneration (Kizil et al., 2014). Lastly, positional mapping of candidate SNPs within a 10-kb window of a gene resulted in the identification of *VIT*, *STRN*, and *HEATR5B* genes for visual network-SC (Extended Data Fig. 3-1). Two intronic SNPs within *STRN* (rs2003585, rs2691112) were also included in the 95% credible causal SNP set during fine-mapping with a posterior probability of being causal (PIP) of 0.14 and 0.12, respectively (see Extended Data Fig. 3-2 for all SNPS in the 95% credible sets identified by fine-mapping).

10.1523/ENEURO.0242-22.2023.f3-1 Extended Data Figure 3-1 FUMA gene mapping based on positional location, eQTL association and/or chromatin interactions in cerebral cortex tissue. “Ensg = ENSG ID. Symbol = gene symbol. Chr = chromosome. Start = starting basepair position of the gene. End = ending basepair position of the gene. Strand = strand of the gene. Type = gene biotype from Ensembl. entrezID = entrez ID (if available). HUGO = HUGO (HGNC) gene symbol. pLI = pLI score from ExAC database. The probability of being loss-of-function intolerant; ncRVIS = noncoding residual variation intolerance score. The higher the score is, the more intolerant to noncoding variation the gene is. posMapSNPs (posMap) = number of SNPs mapped to gene based on positional mapping. posMapMaxCADD (posMap) = maximum CADD score of mapped SNPs by positional mapping. eqtlMapSNPs (eqtlMap) = number of SNPs mapped to the gene based on eQTL mapping. eqtlMapminP (eqtlMap) = minimum eQTL *p*-value of mapped SNPs. eqtlMapminQ (eqtlMap) = minimum eQTL FDR of mapped SNPs. eqtlMapts (eqtlMap) = tissue types of mapped eQTL SNPs. eqtlDirection (eqtlMap) = consequential direction of mapped eQTL SNPs after aligning risk increasing alleles in GWAS and tested alleles in eQTL data source. ciMap (ciMap) = “Yes” if the gene is mapped by chromatin interaction mapping, “No” otherwise. ciMapts (ciMap) = tissue/cell types of mapped chromatin interactions. minGwasP = minimum *p*-value of mapped SNPs. IndSigSNPs = rsID of the independent significant SNPs that are in LD with the SNPs that are mapped to the gene. GenomicLocus = index of genomic loci where mapped SNPs are from. Download Figure 3-1, XLS file.

10.1523/ENEURO.0242-22.2023.f3-2 Extended Data Figure 3-2 95% credible sets as identified by FINEMAP. PIP = posterior inclusion probability (SNPs with PIP > 0.1 in bold), is the posterior probability that this SNP is causal. log10bf = log10 Bayes factors. The Bayes factor quantifies the evidence that the SNP is causal with log10 Bayes factors greater than 2 reporting considerable evidence. mean = marginalized shrinkage estimates of the posterior effect size mean. sd = marginalized shrinkage estimates of the posterior effect size SD. mean_incl = conditional estimates of the posterior effect size mean. sd_incl = conditional estimates of the posterior effect size SD. Download Figure 3-2, XLS file.

### Annotation of identified loci for RSN-FC

We observed two exonic nonsynonymous SNPs in exon 19 (rs2274224, *p *=* *1.771 × 10^−10^) and exon 25 (rs2274223, *p *=* *1.22 × 10^−5^) of the *PLCE1* gene to be associated with limbic network-FC. The *PLCE1* gene encodes for the phospholipase C ϵ1, which mediates the production of two second messengers that regulate cell growth, differentiation, and gene expression (Rao et al., 2017). The high CADD scores (17.35 and 17.48, respectively) suggest deleteriousness of these two exonic nonsynonymous SNPs. rs2274224 was also among the FINEMAP 95% credible set of six SNPs within *PLCE1*, although the intronic rs10786156 had the highest probability of being causal (PIP = 0.36). Additionally, four intergenic SNPs within the same locus were located near the *NOC3L* gene.

On chromosome 10, eight SNPs associated with limbic network-FC were mapped to the *CYP2C8* gene based on eQTL mapping (Fig. 3*b*). Expression of *CYP2C8* results in an enzyme important for drug metabolism (Backman et al., 2016). One of CYP2C8 substrates, the nonselective monoamine oxidase inhibitor phenelzine, is known to target the nervous system and is clinically prescribed as treatment for major depressive disorder (Q. Wang et al., 2019). A large body of research has verified the association between major depressive disorder and changes in limbic network functional connectivity, as well as with other RSNs (for a meta-analysis, see Kaiser et al., 2015).

The annotation of SNPs in the locus that was shared between frontoparietal and somatomotor network-FC revealed only intergenic candidate SNPs (enrichment = 2.15, *p *=* *5.09 × 10^−9^), which convolutes biological interpretation but is a common observation for complex traits (Watanabe et al., 2019). The nearest genes to the candidate SNPs in this locus were *PAX8* and *IGKV1OR2-108* (respectively, 29 and 53 kilobase distance). The likely deleterious SNP rs199993536 (CADD = 19.87) closest to *PAX8* was also the most likely causal SNP in the 95% credible set for both frontoparietal network-FC (PIP = 0.19) and somatomotor network-FC (PIP = 0.42), with a probability of 0.08 that this is the shared causal variant between the two networks. *PAX8* encodes a transcription factor that is considered to regulate the expression of genes important for thyroid development (Blake and Ziman, 2014) and the production of thyroid hormone (Di Magliano et al., 2000). FC within both the somatomotor and frontoparietal network is reduced in individuals with subclinical (Kumar et al., 2018) and clinical hypothyroidism (Singh et al., 2015).

### Default mode network-FC genes associated with Alzheimer’s disease

We next performed gene-based GWAS for the FC and SC within every RSN (Fig. 4). We detected two Bonferroni-corrected genome-wide significant genes additional to the mapped genes described above by combining association statistics from neighboring variants within a single gene (Extended Data Fig. 4-1). Visual network-FC was associated with *APOC1* (*z *=* *5.15, *p *=* *1.31 × 10^−7^), and for default mode network-FC *APOE* was found to be associated (*z *=* *5.13, *p *=* *1.43 × 10^−7^). *APOC1* and *APOE* are both located within the 19q13.2 locus and are well-known risk factors for Alzheimer’s disease (Emrani et al., 2020).

10.1523/ENEURO.0242-22.2023.f4-1 Extended Data Figure 4-1 Association *p*-values of all genome-wide significant (GWS) genes for RSN-FC/RSN-SC. Displayed are genes significant after Bonferroni correction for the number of genes and the number of traits tested. Entrez ID = Entrez ID of gene. CHR = chromosome. Start = start position of the gene in base pairs. Stop = end position of the gene in base pairs. *N* SNPs = number of SNPs in gene. *N* Param = number of relevant parameters used in the model. *N* = per gene sample size; Z = Z-value for the gene, based on its *p*-value; P = SNPwise mean *p*-value (model that uses sum of squared SNP Z-statistics as test statistic). Download Figure 4-1, XLS file.

10.1523/ENEURO.0242-22.2023.f4-2 Extended Data Figure 4-2 Bivariate local genetic correlations from LAVA between summary statistics of Alzheimer’s disease and default mode network-FC. Locus = locus number. chr = chromosome of locus. start = basepair start position of the locus. stop = basepair end position of the locus. n.snps = number of snps within the locus. n.pcs = number of PCs within the locus. phen1 = one of the two phenotypes involved in the bivariate *r*_g_. phen2 = one of the two phenotypes involved in the bivariate *r*_g_. rho = local genetic correlation. rho.lower and rho.upper = 95% confidence interval of rho. r2 = proportion of genetic signal for phen1 that is explained by phen2. r2 lower and upper = 95% confidence interval of r2. ci.lower and ci.lower = 95% confidence interval for partial correlation coefficient. P = *p*-value for local *r*_g_. Download Figure 4-2, XLS file.

10.1523/ENEURO.0242-22.2023.f4-3 Extended Data Figure 4-3 Local *r*_g_ between default mode network-FC and Alzheimer’s disease as performed in LAVA. Only loci that passed the univariate *h^2^* threshold (*p *<* *1 × 10^−4^) were tested for bivariate *r*_g_, resulting in the Bonferroni-corrected significance threshold represented by the red line. Significant loci are visualized with their *r*_g_ estimate. Within these loci, global functional connectivity strength (FC) did not show significant univariate *h^2^* and could therefore not bias these results. Download Figure 4-3, EPS file.

10.1523/ENEURO.0242-22.2023.f4-4 Extended Data Figure 4-4 Results of MAGMA’s gene-set analysis for FC/SC within RSNs in all curated and gene-ontology gene-sets from MSigDB (sets C2 and C5). Gene set = set tested to be associated with cerebellar volume gene-based GWAS sumstats. *N* genes = number of genes used in the analysis. Beta = effect size. Beta SD = standardized effect size. SE = standard error. P = *p*-value. Download Figure 4-4, XLS file.

In order to determine whether there is genetic overlap between Alzheimer’s disease (Wightman et al., 2021) and default mode network-FC, we performed local genetic correlation (*r*_g_) analysis (see Materials and Methods; Extended Data Fig. 4-2). For default mode network-FC, we detected two loci on chromosome 12 (BP 64,403,858–66,114,643) and 19 (BP 45,040,933–45,893,307) which showed significant local *r*_g_ at *p* < (0.05/71=) 7.04 × 10^−4^ with Alzheimer’s disease (Extended Data Fig. 4-3). Given the negligible heritability of global FC in these loci (univariate *p *=* *0.27 and *p *=* *0.01, respectively, whereas *p *=* *1.30 × 10^−5^ and *p *=* *1.62 × 10^−8^ for default mode network-FC) we conclude that these local genetic associations with Alzheimer’s disease are not driven by total brain connectivity. The locus on chromosome 12 showed a positive *r*_g_ ( ρ) between Alzheimer’s disease and default mode network-FC (BP 64,403,858–66,114,643, ρ = 0.69, 95% CI = 0.35–1.00, *p *=* *3.25 × 10^−4^). Interestingly, this locus has been identified in a previous GWAS for hippocampal atrophy, a biological marker of Alzheimer’s disease (Bis et al., 2012). Negative *r*_g_ between Alzheimer’s disease and default mode network-FC was observed in the locus on chromosome 19 (BP 45,040,933–45,893,307, ρ = −0.56, 95% CI = −0.82 to −0.38, *p *=* *9.23 × 10^−9^), indicating that lower default mode network-FC was associated with higher genetic risk of Alzheimer’s disease. Note that this larger defined locus showed weak heritability (*p *=* *0.014) for visual network-FC despite the significance of *APOC1* in the gene-based GWAS, which would make genetic correlation estimates with Alzheimer’s disease unreliable and uninterpretable (Werme et al., 2022). Therefore, Alzheimer’s disease seems to show genetic overlap specifically with default mode network-FC in this locus.

### Looking for biological pathways through gene-set analysis

We looked for convergence of the genetic signal for RSN-FC/RSN-SC onto 7252 MSigDB (Liberzon et al., 2011) pathways using MAGMA gene-set analysis, a useful method for further functional interpretation (Uffelmann and Posthuma, 2020). We conditioned our analyses on the gene-based GWAS summary statistics for global FC/SC in an effort to capture RSN-specific pathways. Five pathways showed an association with four RSNs after Bonferroni correction for the number of pathways tested per trait (Extended Data Fig. 4-4 displays the associations of all pathways tested for all traits). These included blood vessel morphogenesis (GO, *p* = 3.30 × 10^−6^) and vasculature development (GO, *p *=* *4.94 × 10^−6^) pathways for limbic network-SC, the Parkinson’s disease pathway (KEGG, *p *=* *2.10 × 10^−6^) for somatomotor network-SC, the pathway for positive regulation of mesenchymal cell proliferation (GO, *p *=* *2.64 × 10^−6^) in default mode network-FC, and the pathway for negative regulation of histone methylation (GO, *p *=* *5.23 × 10^−6^) for the dorsal attention network-FC. These five pathways did however not survive further Bonferroni correction for the number of traits tested [α = (0.05/7246)/14 = 4.93 × 10^−7^]. Therefore, it cannot be concluded that these biological processes are involved in the genetics of FC and SC within RSNs.

### Examining overlap between structure and function per RSN through genetic correlations

As SC strength has been noted to correlate with FC strength on the phenotypic level (Mollink et al., 2019), we sought to investigate the correlations between FC and SC properties within each RSN on a genetic level. Genome-wide genetic correlations (*r*_g_) were estimated using SNP-based summary statistics (Fig. 5). We observed no nominally significant genome-wide *r*_g_s between SC and FC in any of the RSNs (Extended Data Fig. 5-1). Genome-wide *r*_g_ estimates ranged from −0.19 (SE = 0.15, *p *=* *0.19) in the dorsal attention network and 0.23 (SE = 0.23, *p *=* *0.30) in the frontoparietal network.

10.1523/ENEURO.0242-22.2023.f5-1 Extended Data Figure 5-1 Global genetic correlations between summary statistics of RSN-FC/RSN-SC in LDSC and genomic SEM. *r*_g_ = global genetic correlation. SE = SE of *r*_g_; Z = z-score of *r*_g_. P = *p*-value for *r*_g_. h2 obs = observed scale h2 for trait 2. h2 obs SE = SE of observed scale h2 for trait 2. h2 int = single-trait LD Score regression intercept for trait 2. h2 int SE = SE of single-trait LD score regression intercept for trait 2. gcov int = cross-trait LD score regression intercept. gcov int SE = SE of cross-trait LD score regression intercept. Download Figure 5-1, XLS file.

Strongly localized or opposing local *r*_g’_s possibly may go undetected, since genome-wide *r*_g’_s are an average of the shared genetic association signal across the genome. We examined whether such relationships between SC and FC within any given RSN exist by performing local *r*_g_ analysis (Werme et al., 2022), although we did not identify any significant *r*_g_ on a locus level either (Extended Data Table 1-1).

### Genome-wide and local genetic correlations within the functional and structural domain

We examined the shared genetic signal across RSNs within the same domain by conducting genome-wide *r*_g_ analyses (Fig. 6; Extended Data Fig. 5-1). For functional connectivity strength within the default mode network and within the ventral attention network, a significantly shared genetic signal was observed after Bonferroni correction (*r*_g_ = 0.52, SE = 0.16, *p *=* *1.00 × 10^−3^). This association was not driven by global FC as neither default mode network-FC nor ventral attention network-FC were genetically correlated with global FC (*r*_g_ = 0.19, SE = 0.18, *p *=* *0.29; *r*_g_ = 0.26, SE = 0.19, *p *=* *0.18, respectively). Note that this positive *r*_g_ does not imply simultaneous functional activation of these two RSNs or their involvement in similar cognitive tasks (which would contradict previous research, Menon and Uddin, 2010), but suggests that variants that influence default mode network-FC generally tend to influence ventral attention network-FC in the same direction.

For the structural connectivity property, we observed multiple significant genome-wide *r*_g’_s (*p *<* *1.19 × 10^−3^) across RSNs, although many of these were also correlated with global SC (Extended Data Fig. 5-1). To determine whether genetic overlap of structural connectivity across RSNs could be accounted for by global SC, we used genomic SEM to compute residual *r*_g_ estimates across RSN-SC while taking global SC into account (see Materials and Methods). As none of the residual *r*_g_ estimates remained significant, we conclude that global SC likely accounts for the observed genetic overlap across RSN-SC.

We extended our investigation into shared genetic signal across RSNs beyond the global to the local scale. Eighteen loci showed Bonferroni corrected significant *r*_g_s when comparing RSNs within the functional domain (Table 1). These were all highly positive (mean ρ = 0.84, SD = 0.09) and were not confounded by global FC. When comparing RSNs within the structural domain, local *r*_g_ analysis revealed only one positively correlated locus between dorsal attention network-SC and frontoparietal network-SC (chr15:39238841-40604780, local *r*_g_ ( ρ) = 0.85, *p *=* *9.51 × 10^−7^; Table 1). A complete overview of local *r*_g_ results can be found in Extended Data Table 1-1.

**Table 1:** Local genetic correlations across RSNs within the functional (FC) and structural (SC) domains

Chr	Start	Stop	RSN 1	RSN 2	ρ	95% CI		*p*-value
1	2,215,496	2,983,519	FC SMN	FC VN	0.77	0.47	1.00	3.39 × 10^−5^
1	18,427,821	19,238,924	FC DMN	FC FPN	0.72	0.45	1.00	9.48 × 10^−6^
1	211,082,893	212,347,582	FC VAN	FC SMN	1.00	0.74	1.00	1.75 × 10^−7^
2	113,930,669	115,203,835	FC FPN	FC SMN	0.88	0.64	1.00	3.42 × 10^−7^
2	207,726,595	208,674,588	FC FPN	FC VN	0.97	0.72	1.00	1.02 × 10^−6^
5	4,636,543	5,828,694	FC DMN	FC DAN	0.73	0.47	1.00	2.72 × 10^−5^
5	68,006,994	71,468,651	FC VAN	FC SMN	0.79	0.53	1.00	2.05 × 10^−5^
5	75,959,516	77,290,255	FC DMN	FC DAN	0.91	0.65	1.00	3.42 × 10^−6^
6	10,416,551	11,790,671	FC VAN	FC SMN	0.83	0.55	1.00	2.49 × 10^−5^
7	50,894,509	51,951,647	FC LN	FC VN	0.88	0.57	1.00	5.12 × 10^−6^
8	64,215,359	66,018,204	FC DMN	FC VN	0.86	0.59	1.00	1.09 × 10^−5^
9	93,441,051	94,175,374	FC FPN	FC SMN	0.90	0.61	1.00	1.73 × 10^−5^
9	93,441,051	94,175,374	FC FPN	FC VN	0.87	0.62	1.00	4.58 × 10^−6^
10	89,971,629	91,021,321	FC VAN	FC VN	0.96	0.67	1.00	1.23 × 10^−6^
15	39,238,841	40,604,780	SC DAN	SC FPN	0.85	0.53	1.00	9.51 × 10^−7^
17	13,648,447	14,508,610	FC DMN	FC LN	0.89	0.69	1.00	3.50 × 10^−9^
18	2,839,843	3,722,828	FC DMN	FC DAN	0.70	0.45	1.00	2.66 × 10^−5^
19	17,045,964	17,750,518	FC LN	FC DAN	0.73	0.47	1.00	2.34 × 10^−6^
19	17,045,964	17,750,518	FC DMN	FC DAN	0.79	0.53	1.00	1.43 × 10^−5^

Loci with Bonferroni-corrected significant [*p* < (0.05/774=) 6.46 × 10^−5^] *r*_g_ (ρ with lower and upper limit of 95% confidence interval) between RSN-FC or RSN-SC as performed in LAVA. Within these loci, global FC or SC did not show significant univariate *h*^2^ or *r*_g_ with either of the two RSNs. See Extended Data Table 1-1 for all statistics. SMN = somatomotor network, VN = visual network, DMN = default mode network, FPN = frontoparietal network, VAN = ventral attention network, DAN = dorsal attention network, LN = limbic network.

### Lead SNP validation and polygenic score prediction

We examined the replicability of the discovery lead SNPs as defined in FUMA in our holdout sample (*N*_FC_ = 3408; *N*_SC_ = 3412). From these six lead SNPs, we estimated to replicate three (exact 3.99) at α = (0.05/n lead SNPs per trait) in our holdout sample given their winner’s curse corrected effect size and the sample sizes of the discovery and replication samples. Observations showed three discovery lead SNPs to be significant (Extended Data Fig. 2-4). Extended Data Figure 2-5 shows the probability distributions for all six discovery lead SNPs to be significant at increasing replication sample sizes.

Additionally, the variance that could be explained in RSN-FC/RSN-SC by polygenic scores based our GWAS associations was considered. In PRSice-2, the summary statistics of the discovery GWAS were used to find the best *p*-value threshold for polygenic scores in the target set. The application of this optimal prediction model in our validation set explained on average 0.28% and 0.35% of the variance across RSN-FC and RSN-SC, respectively (Extended Data Fig. 2-3). Note that this *R*^2^ value is on average 2.75% (SC) to 6.89% (FC) of the $h_{SNP}^{2}$, which is a comparable with other studies with relatively small sample sizes and is expected to climb close to $h_{SNP}^{2}$ with increasing sample sizes (Choi et al., 2020).

## Discussion

Mapping the genetic components of resting state networks (RSNs) may provide insight into the etiology of brain function and brain disorders. RSNs are typically defined using functional connectivity (FC) patterns across brain regions, and structural connectivity properties (SC) between these regions correlate with FC across RSNs (Gu et al., 2021). The aim of this study was to gain more insight into the genetic underpinnings of structural and functional connectivity properties (SC; FC) within a framework that respects the brain’s hierarchical functional architecture. With the use of GWAS and annotation we observe that genetic variation in RSN-FC (e.g., limbic network-FC and default mode network-FC) impacts biological processes related to brain disorders (major depressive disorder and Alzheimer’s disease, respectively) that have previously been associated with FC alterations in those RSN. We further identify genes for visual network-SC that are involved in axon guidance and synaptic functioning. The genetic component of RSNs overlaps mostly within the functional domain, whereas less overlap is observed within the structural domain and between the functional and structural domains.

For FC within RSNs (RSN-FC), we detect biologically interpretable results for the default mode and limbic network-FC. For default mode network-FC, we observe *APOE* as a genome-wide significant gene. The default mode network is hypothesized to relate to Alzheimer’s disease through the role of default mode network-FC in memory consolidation (Fox and Raichle, 2007) and through cortical atrophy spreading over default mode network regions over time (Seeley et al., 2009). Here, we complement earlier phenotypic observations that link Alzheimer’s disease to default mode network-FC (Chiesa et al., 2017) by now also showing genetic correlations in two loci between Alzheimer’s disease and default mode network-FC. Functional follow-up would be necessary to investigate how the variants and genes in these loci affect default mode network-FC. The limbic network is commonly known for its involvement in emotion regulation, episodic memory, and action–outcome learning (Rolls, 2019) and has been associated with mood disorders such as major depression disorder and bipolar depression (Liu et al., 2019). The genes *PLCE1*, *NOC3L*, and *CYP2C8* were related to limbic network-FC, all of which have been noted to have a relationship with major depressive disorder (Shi et al., 2012; Q. Wang et al., 2019; Kanders et al., 2020). A previous study investigating the role of *PLCE1* in major depressive disorder patients has demonstrated an association with antidepressant remission in female patients, together with other genes within the calcium/calmodulin-dependent protein kinase pathway (Shi et al., 2012). *NOC3L* eQTLs in the cerebellum and nucleus accumbens have previously been demonstrated to associate with depression severity and antidepressant response (Kanders et al., 2020), and one of the substrates of CYP2C8 is clinically prescribed as treatment for major depressive disorder (phenelzine; Q. Wang et al., 2019). These results seem to suggest that major depressive disorder and antidepressant response involve processes that are impacted by genetic variation in limbic network-FC.

We also find evidence of shared genetic signal in FC across different RSNs. Specifically, we observe a genetically correlated and common genome-wide significant locus for both somatomotor and frontoparietal network-FC near *PAX8*. *PAX8* regulates multiple genes involved in the production of thyroid hormone (Di Magliano et al., 2000), an interesting result considering that both somatomotor and frontoparietal network-FC have been linked to (subclinical) hypothyroidism (Singh et al., 2015; Kumar et al., 2018). Additionally, we detect genetically correlating loci between all RSN-FC and a genome-wide genetic correlation between ventral attention and default mode network-FC. The ventral attention network supports salience processing (Kim, 2010), whereas the default mode network includes areas widespread over the brain and supports emotional processing, self-referential mental activity, and recollection of prior experiences (Raichle, 2015). Increased FC within these two RSNs has been associated with bulimia nervosa (Domakonda et al., 2019) and contributes to episodic memory retrieval (Kim, 2010). Altogether, the shared genetic underpinnings of different RSN-FC that we present here could give a possible explanation how multiple disorders are associated with more than one RSN.

We report considerable heritability estimates for RSN-SC (ranging from 10.00% to 15.40%) and identify nine genes that suggest a role for synaptic transmission in the genetics of visual network-SC. For example, *STRN* encodes for a calmodulin-binding protein that is mostly found in dendritic spines playing a role in Ca^2+^ signaling (Moqrich et al., 1998), the transcript of *FAM175B* is a component of a deubiquitylation enzyme complex that has been suggested play a role in synaptic transmission and synaptic plasticity (DiAntonio and Hicke, 2004), and *SEMA3A* is known as an axonal guidance gene during development (Zhou et al., 2008). The SEMA3A protein has been shown to be upregulated in schizophrenia patients and is suggested to contribute to the developmentally induced impairment of synaptic connectivity in the disorder (Eastwood et al., 2003). Visual network functional hyperconnectivity has been observed in schizophrenia (Damaraju et al., 2014; Ford et al., 2015) and related to visual hallucinations (Ford et al., 2015), but future studies should investigate the equivalent SC component in more detail given our findings.

When investigating the genetic relationship between SC and FC within each RSN, we find no significant genome-wide or local genetic correlations. Since the estimation of genetic correlations is dependent on sample size and the heritability estimates of both traits (Bulik-Sullivan et al., 2015), studies with higher power are needed to examine the robustness of these correlation estimates. Future studies could additionally incorporate recent insights that indirect structural connections supporting direct functionally connected regions complicate simple structure to function mapping (Suárez et al., 2020). Our study focused on direct structural connections within RSNs. The possibility that the genetics of RSN-FC overlap with that of indirect pathways that structurally connect brain regions within RSNs via a route beyond the borders of that RSN could therefore be subject to future research. Another possibility is that RSN-FC genetically correlates with RSN-SC if alternatively defined. Here, we have used two metrics to measure properties of connectivity that are most often used in neuroimaging studies and have well established relevance to neuropsychiatry (Buckholtz and Meyer-Lindenberg, 2012; de Lange et al., 2019). These metrics come however with their own limitations (see Kelly et al., 2012; Jones et al., 2013) and alternative metrics that have been used to measure connectivity are the number of streamlines (Jones et al., 2013), index of axonal density (Fieremans et al., 2011), time dependent efficacy of interactions between brain regions (Friston, 2011), or proxy connectivity measures such as morphometric similarity metrics (Seidlitz et al., 2018).

Several limitations must be considered while interpreting our results. First, the definition of RSN used in this univariate GWAS study reduces voxel-level diffusion and functional information to one phenotype by averaging potentially variable connectivity patterns. This could unequally affect more variable higher-order RSNs compared with less variable unimodal RSNs, which would lead to differential statistical power across the RSNs studied here (Helwegen et al., 2023). This could explain why the most significant results are observed in the visual and somatomotor network. Second, it is known that rsfMRI measures are subject to motion distortion, which raises the possibility of differences in measurement error between RSN-FC and RSN-SC. However, given our stringent preprocessing and quality control to enable noise minimization and additional use of rsfMRI-specific covariates in GWAS, we were able to find heritability estimates for RSN-FC that are concordant with previous studies (Roelfs et al., 2022). Third, although UK Biobank provides genetic and uniform MRI data at unprecedented sample sizes, it is evident that even larger sample sizes are needed for discovering the often small genetic effects of polygenic traits (Visscher et al., 2017). The null results observed for some RSN-FC/SC GWAS, partitioned heritability and gene-set analyses might be explained by the multiple comparison correction for the number of phenotypes analyzed, in conjunction with insufficient statistical power. Fourth, some other sample characteristics, such as the European ancestry, age-class and socioeconomic status of subjects, may limit the generalizability of our findings. While we corrected for age and Townsend deprivation index (a proxy of socio-economic status) in our GWAS to reduce this bias, larger and more diverse imaging-genetics datasets are undoubtedly needed.

This study examines the genetic architecture of RSNs, structurally and functionally. We observe several genetic effects for RSNs that highlight relevant biological processes for brain connectivity and related brain disorders. The complexity of structure-function coupling within RSNs is illustrated by the observation that, despite genetic overlap of RSNs within the functional domain, genetic overlap is less apparent within the structural domain and between the functional and structural domains. Altogether, this study advances the understanding of the complex functional organization of the brain and its structural underpinnings from a genetics viewpoint.

## Synthesis

Reviewing Editor: Christoph Michel, Universite de Geneve Decisions are customarily a result of the Reviewing Editor and the peer reviewers coming together and discussing their recommendations until a consensus is reached. When revisions are invited, a fact-based synthesis statement explaining their decision and outlining what is needed to prepare a revision will be listed below. The following reviewer(s) agreed to reveal their identity: Elvisha Dhamala, Varun Warrier. Note: If this manuscript was transferred from JNeurosci and a decision was made to accept the manuscript without peer review, a brief statement to this effect will instead be what is listed below.

The reviewers evaluated the manuscript generally positively. However, they ask for a few modifications and clarifications that could be handled in a revision.

One aspect concerns the summarizing of FC and SC in 7 cortical networks only, thereby risking the possibility that opposite effects within these networks could lead to non-significant results when combining them. While creating GWAS would be a lot of additional work, one reviewer suggested to not actually run GWAS but expand the analysis by testing genetic correlations with the expanded parcellation scheme, which is more feasible. The editor agrees with this suggestion.

We below add all the comments of the reviewers and ask the authors to carefully consider the comments and specify how these comments have been taken into account in the revised manuscript.

Reviewer 1:

In this manuscript, Tissink and colleagues have examined how different genes are related to structural and functional connections within the brain. While the work presents an interesting set of results, I do have some concern regarding the current set of analyses performed and the manuscript overall.

In their analyses, authors have summarised data from a 250 region parcellation into data representing FC and SC within 7 cortical networks. While there is nothing wrong with this per se, summarising FC and SC to a single value per network is likely to result in a huge loss of signal as that’s basically reducing information from tens of thousands of voxels into a single value. This is especially likely to affect higher-order association networks which are more spatially disjointed and are more likely to have higher variability in their FC and SC profiles. As an example, an association network may include some regions in one part of the brain that exhibit strong positive functional connections and a different set of regions that exhibit strong negative functional connections amongst themselves. By summarising the connectivity to a single value, meaningful information about the variance of connections within a region is lost. This may also explain why most of the significant results in Figure 1 were found in unimodal somatosensory areas which are likely to have less variability in their functional and structural connections within the network. I would recommend expanding the current analyses to consider this variability.

In the GWAS analyses, authors have only considered within-network associations and not between-network associations. However, they later go on to consider genome-wide genetic correlations in those associations between networks. Instead of studying the correlations between networks, why not just consider the associations with between network connectivity? This would provide more insight into whether specific genes are contributing to functional and structural connections between networks.

In the abstract, authors state that “genetic variation in RSN-FC impact biological processes related to brain disorders that have previously been associated with FC alterations in those same RSNs.” While this statement is somewhat justified by their findings, it is extremely difficult to comprehend and could be re-phrased to communicate the findings more clearly.

Authors use RSN-SC throughout the manuscript, which is typically not a standard term as “RSN” is only used in reference to functional data. Perhaps the authors could define this more clearly in their introduction.

Authors have defined structural connectivity to be the average FA for streamlines within a region. However, this is not a typical definition of SC (which often represents some measure of streamline count). Moreover, as the authors state, “FA is a robust measure of white matter integrity and has been found to be sensitive to changes in connectivity", again highlighting that FA doesn’t actually represent connectivity but is rather just sensitive to changes in connectivity. To prevent misleading the readers, I recommend using a different term instead of SC throughout the manuscript.

Finally, authors have used many acronyms throughout the manuscript that they have not defined making the manuscript very difficult to read and understand (e.g. LD, BRISC, SNP, CADD, eQTL, eEF1A, MAGMA, FUMA).

Reviewer 2:

Tissink and colleagues conduct a series of analyses to investigate if there is shared genetics between the functional and structural components of resting state networks (RSN). They use the Yeo-Krienen (please note, Thomas Yoe and Fenn Krienen are co first-authors on this paper, so it will be nice to refer to these networks as Yeo-Krienen and not just Yeo networks) RSN and then calculate the mean FA and average correlation between the activation signals to extract structural and functional metrics in these networks, respectively. They then proceed to conduct GWAS, genetic correlations, and identify biological signatures by annotating the variants and identifying genes. The analyses were conducted using standard GWAS framework and have been done well. I have a few comments.

1. Variant interpretation - Although I am appreciative of the fact that the authors have tried to interpret the significant loci using non-synonymous exonic variants, given that there are so few significant variants, I’d like the authors to use multiple approaches to identify variants and genes. Linking variant to genes is non-trivial. Some suggested approaches would be finemapping of the variants, and using methods such as SMR and Hi-C contact to link fine-mapped variant to gene.

2. I’d like the authors to also to discuss the fact that measuring connectivity is tricky. Here, they have used two metrics to measure connectivity in structural and functional networks. However, there are other phenotypes that can be proxy for connectivity (for example, morphometric similarity metrics).
