# Supplementary figures and images for: The Genetic Architectures of Functional and Structural Connectivity Properties within Cerebral Resting-State Networks
Source: eNeuro. 2023 Apr 7;10(4):ENEURO.0242-22.2023. doi: 10.1523/ENEURO.0242-22.2023 (PMC10089056; doi:10.1523/ENEURO.0242-22.2023)

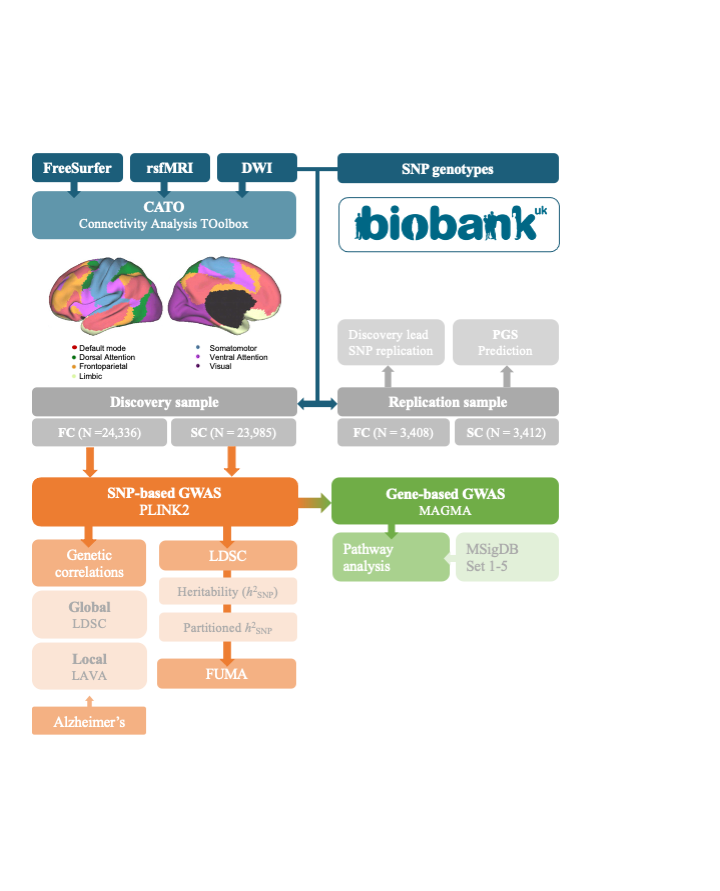

Supplement: Extended Data Figure 1-3 — Flowchart of methods involved in the current study. Functional and structural connectivity (FC/SC) within resting-state networks (RSN) as defined by Yeo et al. (2011) were obtained similarly as previously described (Wei et al., 2019). SNP-based and gene-based GWAS and in silico follow-up were performed on a discovery sample and were validated in a replication sample. rsfMRI = resting-state functional magnetic resonance imaging, DWI = diffusion weighted imaging, SNP = single nucleotide polymorphism, FC = functional connectivity, SC = structural connectivity, GWAS = genome-wide association study, LDSC = linkage disequilibrium score regression, LAVA = local analysis of [co]variant annotation, FUMA = functional mapping and annotation, MAGMA = multimarker analysis of genomic annotation, MsigDB = molecular signatures database, PGS = polygenic score. Download Figure 1-3, TIF file. [file enu-eN-NWR-0242-22-s20.tif]
